# Psychosocial challenges and coping strategies among people with minority gender and sexual identities in Zambia: health promotion and human rights implications

**DOI:** 10.1080/21642850.2023.2173201

**Published:** 2023-02-06

**Authors:** Mataanana Mulavu, J. Anitha Menon, Chama Mulubwa, Tulani Francis L. Matenga, Hoa Nguyen, Karen MacDonell, Bo Wang, Oliver Mweemba

**Affiliations:** a Department of Health Promotion and Education, School of Public Health, University of Zambia, Lusaka, Zambia; b Department of Psychology, School of Humanities and Social Sciences, University of Zambia, Lusaka, Zambia; c Centre for Infectious Disease Research in Zambia, Lusaka, Zambia; d Department of Population and Quantitative Health Sciences, University of Massachusetts Medical School, Worcester, MA, USA; e Department of Behavioral Sciences and Social Medicine, Centre for Translational Behavioral Science, Florida State College of Medicine, FL, USA

**Keywords:** Minority stress, gender minority, sexual minority, LGBT, sexual orientation, psychosocial, Zambia

## Abstract

**Background:**

Sexual and gender minorities face high levels of stigma, discrimination, and violence. In many countries, they are often criminalized and are at risk of mental health challenges. In Zambia, little is known about the psychosocial challenges and coping strategies of sexual and gender minorities. This study sought to explore psychosocial challenges and coping strategies among sexual and gender minority populations in Lusaka, Zambia to inform mental health and human rights promotion for this population.

**Methods:**

The study used a qualitative phenomenological study design. Data were collected through in-depth interviews with 16 sexual and gender minority participants (lesbian, gay, bisexual, and transgender) and four key informants. The sexual minorities included four lesbian, five gay, and three bisexual participants while the gender minorities included two transgender men and two transgender women. Interviews with gender and sexual minorities were mostly focused on the lived experiences of participants, while those of key informants focused on their work with sexual and gender minorities. Snowball strategy was used to recruit participants, while purposive sampling was used to select key informants. All interviews were recorded and transcribed verbatim. Thematic analysis was carried out with the aid of Nvivo 12 software.

**Results:**

Psychosocial challenges included victimization in the form of threats and physical assault. Stigma and discrimination were experienced in different settings such as healthcare, the workplace, and school. Participants reported having experienced feelings of depression. Rejection from family members was experienced by those who revealed their sexual or gender minority status. Reported coping strategies included social support, self-concealment, listening to music, and substance use.

**Conclusion:**

This study suggests that sexual and gender minorities in Zambia experience various psychosocial challenges related to their sexuality and gender identity. To assist them cope better with the obstacles they experience, improved psychosocial counseling and mental health services are needed.

## Introduction

Sexual and gender minorities, also known as Lesbian, Gay, Bisexual, and Transgender (LGBT) people, experience widespread stigma, discrimination, and violence, and are frequently criminalized in many countries (Carroll & Mendos, 2017). This situation puts them at risk of mental distress and disorders due to social stress (Su et al., 2016; Sun et al., 2020). The social-cultural environment, unfavorable policies, unconducive legal environment, and untrained medical personnel further fuel stigma and discrimination (NASTAD, 2015). They also live in fear of being stigmatized, blackmailed, attacked, raped, jailed, or even killed (UNDP, 2019). Human Immunodeficiency Virus (HIV) is frequently undetected in these populations because they are chronically underserved and may not receive adequate, stigma-free testing opportunities (Fay et al., 2011).

Stigma and discrimination are some of the biggest challenges faced by sexual and gender minorities around the world (Fredriksen-Goldsen et al., 2014; Malta et al., 2020). Stigma and discrimination occurs in different settings, such as healthcare settings (Albuquerque et al., 2016) and the workplace (Christy & Sears, 2011). Sexual and gender minorities are also susceptible to substance use as a coping strategy for the numerous stressors they face (Lewis et al., 2016). Social support is another coping strategy (Simons et al., 2013). Social support, particularly from family members, decreases psychological distress (McConnell et al., 2016).

Sub-Saharan Africa is disproportionately affected by the Human Immunodeficiency Virus/Acquired Immunodeficiency Syndrome (HIV/*AIDS*) pandemic, with the prevalence of HIV among sexual and gender minorities rising (Jin et al., 2021; Mpondo et al., 2017; Ogueji, 2021). As a result, the majority of research on sexual and gender minorities in Africa has concentrated on HIV/AIDS (Djamond et al., 2014). In Zambia, not only do sexual and gender minorities deal with HIV, stigma, and discrimination, but are further marginalized due to the existence of punitive legislation. Further, the self-declaration of Zambia as a Christian nation has been utilized to promote and justify the marginalization of LGBT individuals (NASTAD, 2015; UNDP, 2019). The delivery of mental health services in Zambia is guided by the *Mental Health Act* No. 6 *of* 2019 (The Mental Health Act, 2019). Mental health disorders affect approximately 20 per cent of the population in Zambia. Barriers to mental health care include poor infrastructure, limited human resources, and inadequate funding (Munakampe, 2020). Despite these obstacles, there is a paucity of research on LGBT people in Zambia that explores psychosocial challenges and coping strategies in depth. Although several studies have been conducted on psychosocial challenges and coping strategies among sexual and gender minorities in developed countries such as the United States, these findings cannot be translated to other settings or contexts like Zambia, where LGBT individuals are criminalized, thereby limiting their access to appropriate healthcare services.

Therefore, this study explored the psychosocial challenges and coping strategies among sexual and gender minorities in Zambia. The finding from the study shed light on the challenges faced by LGBT individuals in Zambia, and other countries in Sub-Saharan Africa with similar sociocultural settings. These findings could be used to inform the development of LGBT-specific or inclusive mental health and human rights interventions by policymakers and practitioners. The collected data could also provide human rights activists with evidence to support the modification of laws and health promotion/public health approaches that are discriminatory towards LGBT individuals.

**Figure 1.**
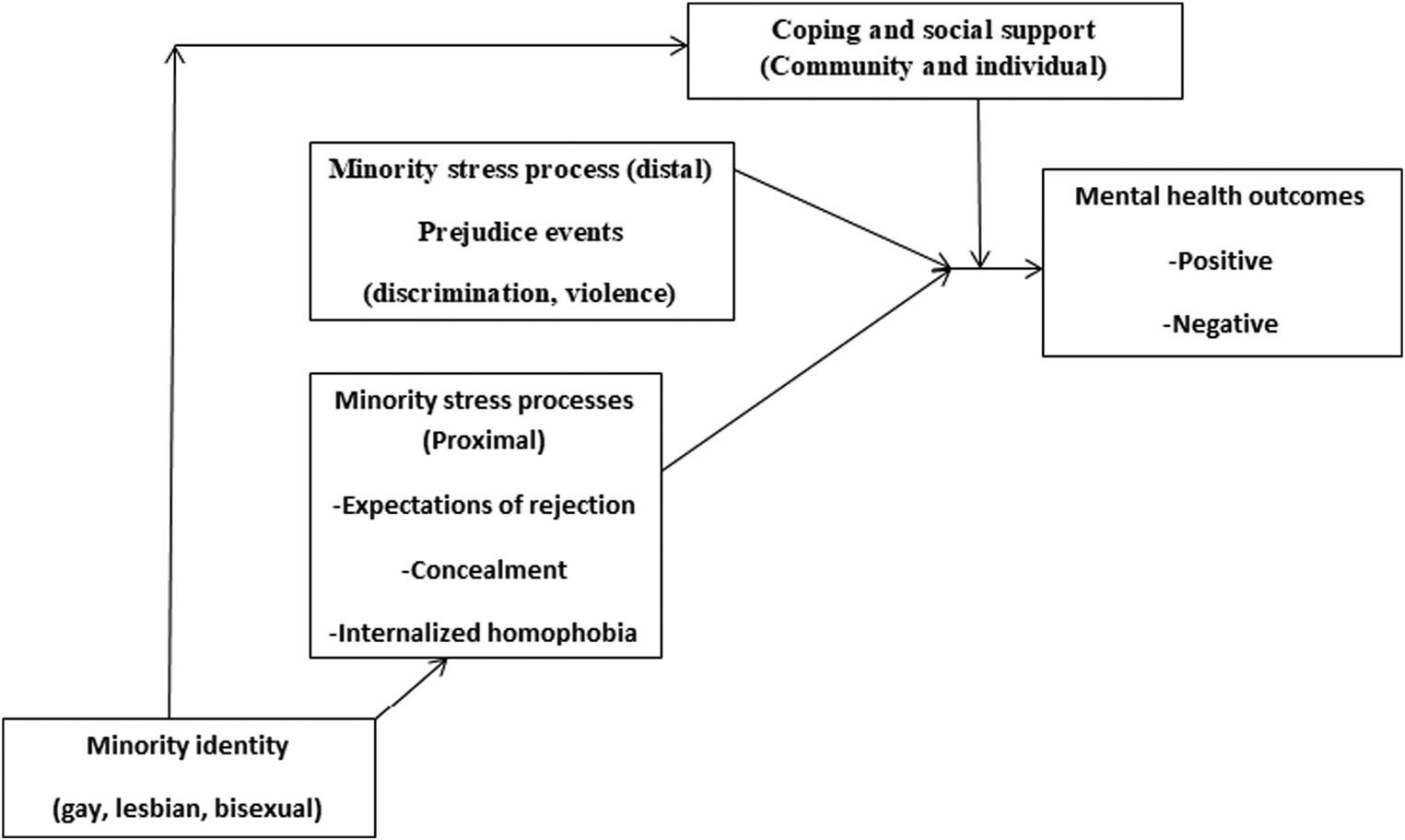
Minority stress process in LGBT populations (adapted from Meyer, 2003).

Meyer’s minority stress theory was adopted to conceptualize this study, create the interview guide, and interpret the results (see Figure 1). Meyer’s minority stress theory explains why poorer mental health is observed among sexual minorities. Minority stressors in form of proximal and distal factors interact, thereby affecting mental health. These minority stressors include experiences of prejudice events, violence, and expectations of rejection, concealment, and internalized homophobia. Public health professionals, public policymakers, and human rights practitioners ought to understand the causes of minority stress to design effective prevention and intervention programmes (Meyer, 2003). Components of the minority stress theory have been applied to the study of alcoholism, depression, and suicidal ideation among sexual and gender minorities (Lea et al., 2014; Baams et al., 2015; Tebbe & Moradi, 2016; Lehavot & Simoni, 2011). This study used the theory by focusing on the component of distal minority stressors to investigate stigma, discrimination, and violence-related issues. It further utilized the component of proximal minority stressors to explore issues around concealment. The component on mental health outcomes was used to explore the emotional and mental well-being of the participants. Lastly, the component on coping and social support was used to investigate coping strategies (Meyer, 2003). This paper addresses the following research questions: (1) What are the psychosocial challenges that LGBT people in Zambia face? (2) What coping strategies are used by LGBT people in Zambia?

## Methods

### Study design and setting

This was a qualitative phenomenological study conducted among LGBT individuals in Lusaka, Zambia, from December 2018 to September 2019. A phenomenological design is used to uncover lived experiences as described by research participants (Cresswell, 2014). The purpose of this design was to describe the lived experiences of sexual and gender minority groups in Zambia. The primary objective was to gain a comprehensive understanding of the psychosocial challenges and coping strategies used by the participants. This method was chosen because it allowed participants to fully narrate their experiences.

### Participants and recruitment

Sixteen participants were recruited in the study using a snowball sampling strategy. Participants were eligible for enrollment if they met the following criteria: (i) resided in Lusaka, Zambia at the time of the study; (ii) self-identified as LGBT; and (iii) were at least eighteen years old or older. Participants also included key informants. A total of four key informants and participants who identified themselves as being lesbian (*n* = 4), gay (*n* = 5), bisexual (*n* = 3) and transgender (*n* = 4) were enrolled in the study. Key informants were recruited purposively, based on their working experience with LGBT populations. The summary of participant information is presented in Table 1.

**Table 1.** Participant characteristics.

Characteristic	n
Age (Mean, SD), years	27.7 (6.5)
Sexual/Gender identity	
Lesbian	4
Gay	5
Bisexual (m)	2
Bisexual (f)	1
Transgender man	2
Transgender woman	2

To recruit participants, the study team worked with organizations working directly with LGBT populations for initial recruitment. These organizations provide various services to sexual and gender minorities, such as HIV services and legal assistance. To recruit participants, the first author (MM) approached a key informant and provided a detailed description of the study. The initial key informant then emailed three additional potential key informants, who informed the LGBT communities about the study and assisted in participant recruitment. The first key informant contributed to the recruitment of six participants, while the remaining two each contributed to the recruitment of two participants. Before the interview, all participants were contacted by phone. A total of 14 individuals were contacted via phone. Ten of the 14 individuals contacted agreed to participate in the study. From the 10 participants who agreed to participate, some of them enabled the research team to recruit six more participants.

### Data collection

Data collection was led by the first author (MM), through face-to-face in-depth interviews. The duration of interviews ranged from 30 to 90 minutes. A semi-structured interview guide was used to direct the flow of the interviews. The discussion guide was developed based on the minority stressors outlined in the minority stress theory, and literature on psychosocial challenges and coping strategies among LGBT individuals (Bockting et al., 2013; Meyer, 2003; Rood et al., 2016). The interviews were conducted in English and covered a wide range of topics, focusing primarily on the participants’ lived experiences as sexual or gender minorities in Zambia. The discussion explored experiences with feelings of depression, stigma, and discrimination, victimization, and rejection. Participants gave responses to questions such as ‘What has life been like being part of a sexual or gender minority group?’ ‘How can you describe your emotional/mental well-being?’ The interview guide also contained questions on coping strategies, substance use, social support, and concealment. Questions such as ‘kindly explain how you are managing being part of a gender or sexual minority group?’ What activities do you engage in during times of stress? Key informants were asked to discuss their experience working with LGBT populations, the challenges the LGBT populations they serve face and the most effective means of addressing these challenges. Among the questions asked was, ‘What challenges have you observed among the LGBT populations you serve?’ All interviews were audio recorded.

### Data analysis

Data were analyzed thematically. Recorded interviews were downloaded onto a computer and transcribed verbatim. Co-authors were provided with transcripts for review and validation. MM led the analysis of the data. Transcripts were read multiple times for familiarization purposes. A codebook was then developed in consultation with the co-authors. Data coding involved matching codes to the segments of data from transcripts that were representative of that code. Coded data were then organized into themes. Thereafter, themes and subthemes were identified. This was done by grouping all the themes that explored a particular subject into one overreaching major theme. This process was carried out with the aid of Nvivo 12 data management software. The development of themes was guided by literature, and the minority stress framework. All collected data were stored securely on a password-protected computer accessible only by the study team. The dependability of the findings was achieved through respondent validation or member checking. Member checking involves comparing the accounts of the researcher and those of the respondents to establish a level of correspondence between the two accounts (Mays & Pope, 2000). To validate the study findings, two of the authors (MM and TFLM) were invited to present study findings at a key population workshop held in September 2019, and received feedback from participants. This was done to ensure the themes were developed accurately and represented the experiences of the participants.

The data collector (MM) has a Master’s degree in Public Health and a degree in Psychology. He is experienced in conducting research and has collected data through interviews in various studies. Data analysis was led by MM and OM. OM has a Ph.D. in social science and health with over 10 years of experience in conducting and analyzing qualitative studies.

### Ethical consideration

Ethical approval for this study was granted by the University of Zambia Biomedical Research Ethics Committee (UNZABREC) (Reference No. 049-08-18). Before the interview, the research study was explained in detail to potential participants. Those who were willing to take part provided verbal consent before participation. To uphold the confidentiality of the participants, no names were recorded. In addition, all data were stored on a password-protected computer accessible only to the study team.

## Results

### Participants’ characteristics

A total of 16 LGBT participants participated in the study, with a mean age of 28 years (range: 18–41) (Table 1). Five of them identified as gay, four of them identified as lesbian, two were bisexual males, and one was a bisexual female. There were four transgender participants with two being transgender men and the other two transgender women.

There were four key informants (see Table 2), each coming from an organization that works with sexual and gender minorities.

**Table 2.** Key informants.

Key Informant	Position	Responsibilities
KI 01	Key population advisor	HIV programing for key populations
KI 02	National project officer	Project management and advocacy
KI 03	Outreach officer	Community outreach activities
KI 04	Director	Project management

### Interview themes

The study participants presented various information that helped in the development of themes and sub themes. The main themes relating to psychosocial challenges and coping strategies and their frequency are presented (see Table 3). The sub-themes, under psychosocial challenges, included feelings of depression, stigma, and discrimination, rejection, and victimization. The sub-themes under coping included music, substance use, self-concealment, and social support.

**Table 3.** Themes and sub-themes.

	Major themes	Subthemes	Illustrative core idea	Frequency
Psychosocial Challenges		Feeling of depression	Experiencing sadness/depression/suicidal ideation/attempts	12
		Stigma and discrimination	Experiencing stigma and discrimination due to being a sexual/gender minority	12
		Rejection	Experiencing rejection from family/friends due to sexual orientation/gender identity	9
		Victimization	Any reference to experiencing terror /violence or threat of violence owing to being a sexual/gender minority	9
	Coping strategies	Music	Listening to music; creating music	8
		Substance use	Alcohol; smoking to reduce the feeling of stress	6
		Self-concealment	Hiding sexual or gender identity from others	7
		Social support	Any reference to having a network of people to turn to in times of need	10

### Psychosocial challenges

Participants in the study reported an assortment of psychosocial challenges, which arose due to society’s negative views and attitudes toward LGBT individuals. Zambian society has a generally negative view of LGBT individuals. These negative views are expressed publicly through various means such as social media. Participants reported how these negative views, along with the experience of prejudiced events, contributed to their psychological distress. The majority of participants reported experiencing depression at least once or on multiple occasions. Negative views of society frequently led to feelings of isolation, suicidal ideation, and suicide attempts.

I stayed like I literally blocked myself from Society. I wouldn’t go out of the house. The only places I would go to is school or maybe church or something like that. I would not mingle with any of my friends, I would not mingle with anyone and I got used to staying in the house because of  …  because of that inner depression I felt like ok I’m not loved by society like they don’t want to see me outside. I would literally stay in the house it was almost like 3 years of me hiding in the house. (Trans woman, 22 years old)

It was found that conforming to what society deemed appropriate behavior, based on one’s gender, caused transgender participants distress. The Zambian society has relatively strict gender norms that are used to guide behavior based on one’s gender. Participants who identify as transgender reported experiencing distress due to difficulties in conforming to societal expectations. One participant reported experiencing depression as a result of the pressure to conform to society’s expectations and his desire to fit in as a transgender man.

Generally most days I experience a bit of depression and it’s not something that one decides to have it just comes because certain times you feel the pressure to blend in with everyone else because certain times you don’t feel like you fit in. (Trans man, 26 years old)

Feelings of depression also manifested through suicidal thoughts and attempts. Participants disclosed that suicidal thoughts had persisted throughout their lives. They discussed how the treatment and perceptions of LGBT individuals by society exacerbated these feelings.

The only person that knows this thing is just you and you’re constantly been bullied, you’re constantly being ridiculed. You don’t feel you belong. You don’t feel you belong, you probably feel you’re better off not been here at all. You’re better off not living at all. The mental stress is so much there. (Gay, 26 years old)

Suicide attempts were also reported as one participant narrated her ordeal after experiencing discrimination from her own family due to her sexual orientation.

The first time I locked myself in our bathroom (…) and I had drunk a bottle of domestos (liquid cleaner) and it didn’t give me the results I wanted but I woke up in hospital. It’s very frustrating I’d rather not wake up than wake up. A failed attempt of suicide is the worst experience ever. (Lesbian, 22 years old)

Additionally, key informants echoed difficulties with suicidal ideation and attempts. Due to the dire situation LGBT people faced in Zambia, suicidal ideation and attempts were reported to be a common occurrence. Some key informants were aware of individuals who had gone as far as taking their own lives.

A lot of community members have killed themselves. I personally know of is it 3. I personally know of 3 that have killed themselves. I think 2 were gay (…) one literally was a doom (poison) situation and another one was where you just start drinking to the point where you kill yourself. Literally someone didn’t just want to live. (KI 02)

### Stigma and discrimination

The majority of participants reported experiencing stigma and discrimination due to their sexual orientation or gender identity. This discrimination was encountered in a variety of settings, and in some instances, it was perpetuated by close family members. The majority of this stigma and discrimination was encountered in schools, the workplace, and healthcare settings. In Zambia, LGBT individuals experience stigma and discrimination in various settings. This stigma and discrimination is often overlooked due to a lack of laws protecting individuals from discrimination based on sexual and gender identity. One participant described how she experienced stigma and discrimination while attempting to access healthcare.

There’s this time one time I went to the clinic and I had a situation and this person wasn’t just, nurse, it was a nurse actually wasn’t really willing to understand my situation cause she thought it wasn’t normal. It was not normal to have that kind of situation and apparently I didn’t get the necessary treatment I was supposed to get and fortunately I met someone who referred me to the right place where I wouldn’t be stigmatised. (Trans woman, 22 years old)

Key informants were aware of the stigma and discrimination experienced by LGBT individuals. They highlighted how stigma and discrimination affected LGBT individuals’ when trying to access healthcare. In some cases, sexual and gender minorities presented a sexually transmitted infection (STI) that made healthcare providers hesitant to provide care due to the nature of the STI.

Sometimes even denied healthcare services because of the condition that they present. So because they had unprotected sexual encounter then they get an STI and that STI is on the anus how are they�� …  you know you’re trying to explain to the healthcare  …  so then the health (healthcare provider) asks you how did you get  …  so at that level, at a more, at a very serious level being denied of services is a serious issue I think. (KI 02)

Some participants reported being discriminated against at learning institutions. They stated how fellow students and lecturers singled them out for abuse, mockery, name-calling, and embarrassment.

At school, there was this particular incident that happened where I almost quit school (…) my lecturer went through the positives which it brings togetherness, it brings understanding. Then he went to the negative side and talked about false image (false representation of reality) and he stopped the whole lesson and he pointed me out as uhmmm  …  he pointed me out as an example of a very good result of false image. (Trans woman, 19 years old)

Some participants described instances of discrimination in the workplace. This type of discrimination manifested itself in their job search efforts as well as employment discrimination. In some cases, discrimination occurred due to employment-related documentation issues. Citizens of Zambia who reach the age of 16 years can obtain a National Registration Card (NRC). Zambian national registration cards include basic information about the cardholder such as name, date of birth, and the sex of a citizen; consequently, transgender people may appear differently than the information on the card in certain instances. Due to employers’ lack of understanding of gender identity issues, employment difficulties may arise.

I was discriminated at a place of work you see because I couldn’t  …  my paperwork did not match how I looked. So because of that my documentation, my NRC (National Registration Card), we needed fingerprints at that point to a certain company that I applied for and because of my sex on the ID card I was denied the job even though I was qualified to work. (Trans man, 26 years old)

The ability to find employment and support oneself was also described by key informants as challenging. Transgender individuals, in particular, were cited as having a difficult time securing employment. Primarily, these obstacles stemmed from how transgender individuals appeared to the hiring manager.

At the community level especially for the transgender people and it’s very difficult for them to fit into society so that also impacts their access to economic empowerment, opportunities, job opportunities because they look a certain way so sometimes employers do not even employ such a person, the moment that they see this person at the interview table and they ask are you he, she or he and from there the chances are slim, so these are some of the issues (KI 01)

In addition to the fact that some participants find it difficult to find employment, others narrated how they lost employment due to their sexual orientation. One participant reported that he lost his job after his sexual orientation became known.

Some people they used to tell me that you’re like gay so you have to stop working. The boss even called me in the office you should stop working we don’t allow such people to be in our whatever [workplace]. (Gay, 40 years old)

### Rejection

The study revealed that most of the participants experienced rejection. It was revealed that family and friends are the most common sources of rejection. Some participants described how coming out to family strained their relationships. Participants reported that their families viewed them as outcasts upon discovery of their minority sexual or gender identity. Family rejection, towards some participants, made them leave home early as teenagers. Some participants reported leaving home due to the discomfort they experienced from their family.

With my dad it kind of took a toll on me because then he cut me off a lot of things, he cut me off, well he cut me off things like I don’t know, things like allowances, luckily I just finished school. The first time I just finished school so it was ok and then he also kind of cut me off from seeing the rest of my family because now I couldn’t go home so it did take a toll on me. (Lesbian, 26 years old)

Rejection has been felt from the day they found out about my orientation so it’s always been there. It’s always been there and because of that I decided to isolate myself completely from my family and that’s why I became independent at a young age and that’s why I left the home when I was about 17 (Lesbian, 22 years old)

Key informants also expressed that sexual and gender minorities experience rejection. Some of them ran programmes that assisted those who have been rejected. Some of these programmes were designed for participants who have been evicted from their homes.

I have helped a lot of people with the programing that we have for instance we have a program called REACT where we go and help people that have been disowned or maybe violated so we try and help them in terms of maybe nutritional support and also to take them to the clinic (KI 04)

### Victimization

The majority of participants reported being victimized due to their sexual orientation or gender identity. According to some participants, this was especially prevalent during their school years. The manifestation of this victimization was in form of bullying. Participants also described being verbally abused frequently. Some participants had also been physically assaulted.

One time I was walking down the road around 19 to 20. I just wanted to get airtime so this blue Hilux with no number plate just pulls up by my side and this man gets out of the car. He strangles me by throat and spits saliva on me and he’s like this is Zambia we’ll kill you if you do not behave like a man (…) people that knew me just by passed me like nothing was happening. Others were laughing and that really scared me because that man would have taken me in his car and no one would have done anything. That man could be stalking me, he could be following me and it’s very difficult for me to go to the police station to report such a person because even just going to the police station and presenting my case is a threat on its own. (Trans woman, 19 years old)

In addition, key informants were aware of the prevalence of victimization among LGBT populations. This victimization was the result of societal prejudice against LGBT individuals in Zambia. Key informants highlighted the effect of victimization on their mental health.

But also the safety and security of this population is highly compromised within the community so some face emotional, physical abuse, some are raped. Some are beaten and also that translates into mental health issues so that’s one of the biggest challenges that they have and they resort to substance abuse to cope with those challenges. (KI 01)

However, one key informant was of the view that despite the widespread victimization of LGBT people, there was a reduction over the years.

So I think these are some of the issues with young gay men. It’s always bullying, mockery and though this was a common narrative when I started this work but now it’s becoming less and less. (KI 02)

### Coping strategies

The findings revealed that participants used various methods to cope with the minority stressors that they experienced. The results below show the different coping mechanisms used by the participants.

### Music

Participants revealed that they used music as a way of coping. Music was used in times of heightened stress or after the experience of a prejudiced event. Participants narrated how they listened to music in stressful situations and how it made them feel better afterwards.

I think one way of coping with stress is usually I love listening to music, love writing music that’s how I kind of like let out all my emotions because everything that I think about if it’s depressing, if it’s hurting me I usually write it down and I make music out of it though no one has heard it apart from my best friend. (Trans woman, 22 years old)

### Social support

Participants pointed out how support from friends helped them deal with the various challenges they went through. They revealed that peer support helped them navigate stressful situations.

Very supportive, they’re always there when I need someone to talk to, they always encourage me. They link me to doctors to help me with my HRT (Hormone Replacement Therapy), yeah things around that. (Trans woman, 19 years old)

Another transgender woman also pointed out how social support had played a significant role in her life.

With my friends I relate well with them and for them knowing that I am a trans woman they kind of encourage me to kind of like upgrade because in our society where you can’t really dress like a woman because they’re going to maybe injure you, try to say some abusive things. And most of my friends have been encouraging me to just open up to the community and see if people are really going to accept me for really who I am. So I think my friends have been a supporting factor in my life. (Trans woman, 22 years old)

### Substance use

As a means of coping with the various minority-related stressors, participants disclosed having used substances such as alcohol and cigarettes. Substance use was a means of forgetting about problems and coping with difficult situations. Substances were primarily used as a means of coping with and relieving themselves of the various stresses that they faced daily.

 … and then when you go out there people are talking about you. You know all those things around you so because you’re stressed all you can think about is let me just go have a drink maybe it will erase all those things. (Trans man, 34 years old)

I drink a lot, I drink heavily. So just to escape that, you feel lots of different emotions every day. (Gay, 26)

Key informants expressed similar sentiments on the reasons why LGBT populations abused substances. They believed that substance abuse was primarily a means of coping with the daily difficulties of belonging to a stigmatized and discriminated gender/sexual minority group.

This is why people [referring to LGBT individuals] abuse substances because [LGBT] people are already are not alright psychologically, you know you can’t deal with things so you’re hoping that the alcohol will handle the situation, you’re hoping that the marijuana will handle the situation. (KI 02)

### Self-concealment

Some participants revealed that they had concealed their sexual orientation or gender identity. One of the main reasons for the concealment was to avoid experiencing society’s negative attitudes towards gender and sexual minorities. Participants revealed that they were more willing to reveal their sexual orientation or gender identity to close friends than family members.

Talking to people about my orientation, no I don’t because society doesn’t accept who I am and the Zambian society is very hostile so I can’t open up freely to people that am like this. (Gay, 23 years old)

Participants indicated that they were more open to their friends and people who were like them. These interactions, engagements, and connections with similar others could also foster an individual’s capacity to cope.

It’s a process, am not yet open. Am open to my friends but not to any other person, if am really convinced that that person is part of our community that’s when I tend to be you know what am also like that. (Gay, 22 years old)

## Discussion

This study contributed to the existing literature by presenting psychosocial challenges and coping strategies in a unique setting. The primary psychosocial challenges faced by sexual and gender minorities are depression that leads to suicidal ideation and attempts, stigma and discrimination, rejection and victimization. The primary coping mechanisms included music, substance abuse, self-concealment, and social support.

For the participants in this study, poor mental health primarily manifested as feelings of depression. Similar to a previous study (Almeida et al., 2009) depression has been shown to affect sexual and gender minorities. Depression affects sexual and gender minorities resulting from persistent victimization from adolescence to adulthood (Mustanski et al., 2016). This study also revealed that depression also manifested itself through suicidal ideation and/or attempts. LGBT individuals experience suicidal ideation and attempts due to the chronic stress of living in hostile social environments (Mereish et al., 2014). Similar to previous research (Mustanski et al., 2010), some participants in this study disclosed suicidal ideation with some respondents also attempting suicide. To reduce these disparities, it is crucial to train more mental health professionals in Zambia to address mental health concerns among the LGBT population. The *Zambian Mental Health Act* No. 6 *of* 2019 stipulates that the minister is responsible for promoting mental health and preventive programmes (The Mental Health Act, 2019). Therefore, it is expected that this obligation should be extended to sexual and gender minorities.

According to the minority stress theory, sexual minorities are victimized due to their identities (Puckett et al., 2016). Participants in our study also reported experiencing victimization, including physical assault. In some cases, law enforcement officers perpetrate victimization where the LGBT communities are subjected to harassment and unjust arrests. Furthermore, in Zambia, when LGBT people are physically assaulted by members of the public, law enforcement officials are reluctant to intervene, and in some instances, they victimize the complainant (UNDP, 2019). Due to the socio-legal environment, victimization and hate crimes against gender and sexual minorities are frequently unreported. In contrast to the majority of studies conducted in developing nations, sexual minorities still face prosecution, and a lack of protective laws (NASTAD, 2015). This necessitates training law enforcement officers on the necessity of combating violence against sexual minorities without prejudice.

Studies demonstrate that stigma and discrimination occur in various contexts, including the workplace (Christy & Sears, 2011). Similarly, this study revealed that sexual and gender minorities faced stigma and discrimination in the workplace. The study revealed that obtaining employment in the workplace was difficult, particularly for gender minorities due to their presentation or physical appearance. This study also revealed that sexual minorities could be fired based on their identity. In turn, this affected their economic situation, further disempowering them. Some participants encountered stigma and discrimination when attempting to access healthcare due to their condition, one of the transgender participants in this study was denied healthcare at a health facility. Seelman et al. (2017), found that transgender people are hesitant to seek medical care due to stigma and discrimination at health centers, which worsens their health conditions. To combat stigma and discrimination in workplaces and health centers, anti-discrimination policies for LGBT individuals seeking employment and health care should be developed. Zambian healthcare providers also require training on the health care needs of LGBT populations.

Another challenge identified by this study was family rejection. Family rejection has been demonstrated by earlier research (Katz-Wise et al., 2016). In some cases, rejection involves disownment or eviction from the family home (Durso & Gates, 2012). Some families have severed communication with their LGBT family member. Negative health outcomes are associated with family rejection, including an increased risk of depression, suicidal ideation, and illicit drug use (Klein & Golub, 2015). When the home environment is more accepting and tolerant, the effects of minority stressors lessen. To combat family rejection, caregivers must be educated on the detrimental effects of rejection on LGBT family members (Ryan et al., 2009).

In this study including Ryan et al. (2009), McConnell et al. (2016), social support was found to be an effective coping strategy because it helped participants better manage their identities. According to Bockting et al. (2013), peer support plays an important role in reducing the psychological distress caused by stigma. It has been suggested that identifying with similar others facilitates the development of a positive in-group identity, promotes a positive self-evaluation process, and provides access to ‘group-level coping,’ all of which have protective effects on the mental health of marginalized individuals (Hendricks & Testa, 2012). This study revealed that, in addition to social support, music was used as a coping mechanism to manage emotionally intense events. This was not a common finding in many previous studies, an indication that it may be necessary to conduct additional research on the effectiveness of music in assisting LGBT individuals to cope with their challenges. In addition, similar to previous research (Lehavot & Simoni, 2011; Rood et al., 2016), this study also found that sexual and gender minorities used substances to cope with stress. Alcohol abuse was predominantly used to manage negative emotions which may explain why LGBT people are susceptible to substance use.

Lastly, similar to a study conducted by Hoy-Ellis (2016), the use of concealment or non-disclosure as a coping mechanism emerged in this study. Concealment was as a result of avoiding rejection from friends and family members or out of fear that one's LGBT identity would be revealed to the general public. Unfortunately, prolonged concealment leads to an increase in stress, resulting in a rise in psychological distress (Meyer, 2003). The hostile socio-legal climate in Zambia encourages concealment, resulting in further marginalization (UNDP, 2019). This situation disadvantages LGBT individuals as they are less likely to access services that would be to their advantage (Meyer, 2003). Therefore, it is necessary to encourage LGBT individuals to form support groups in which they are free to discuss their various challenges. It is important to note that this study revealed that concealment is typically difficult for transgender individuals, as they prefer to dress and act in the manner of the gender they identify with.

Despite the aforementioned difficulties faced by LGBT populations in Zambia, health promotion practices have sometimes excluded sexual and gender minorities (Mule et al, 2009). Published research in Sub Saharan Africa, including Zambia, has shown the negative impact of the HIV pandemic and reduced use of HIV and other STI health services among sexual and gender minorities (Balogun et al., 2020; Gamariel et al., 2020; Magesa et al., 2014; Pilgrim et al., 2019; Wanyenze et al., 2016; Wirtz et al., 2014). Beyond HIV and AIDS, gender minorities continue to experience a variety of sexual/gender identity-related obstacles, including mental health issues (Bockting et al., 2013; Mustanski et al., 2010; Rood et al., 2016).

The right to the highest attainable standard of health for all is a fundamental human right of significant importance (OHCHR, 2008). This right encompasses health promotion, which seeks to empower individuals to increase their control over their health (Kumar & Preetha, 2012). Consequently, countries are encouraged to engage in health promotion for all citizens (Tasioulas & Vayena, 2016). The right to healthcare, including mental health services, should be extended to all individuals, including those who face structural or human rights discrimination (Arulkumaran, 2017). To ensure safe access to services, it is essential to evaluate criminalizing laws that exacerbate discrimination against sexual and gender minorities (Ferguson et al., 2019). The human rights framework provides a universal framework to promote justice in public health, and improve marginalized groups’ access to desperately needed psychosocial health interventions (Meier, 2017).

### Strengths and limitations

The strength of this study is that it recruited sexual and gender minorities that fall into different groups, hence providing diverse information. The limitation of this study is that it was conducted in Lusaka with a small sample size. Therefore, the views may not represent the experiences of other LGBT populations outside of the study setting. Another limitation is that it did not explore differences in the experience of minority stress among sexual and gender minorities.

### Recommendations

There is a need to improve access to mental health services for LGBT individuals, through the development of friendly services that cater to their psychosocial health needs, while also taking into consideration the legal context around LGBT issues in Zambia.

There is need to integrate psychosocial services within the healthcare system that should be aimed at addressing the psychosocial needs of LGBT populations.

Mental health providers should be trained on the psychosocial challenges LGBT individuals face, and how best to promote adaptive coping strategies for the challenges they go through.

## Conclusion

This study was conducted to gather information on the psychosocial challenges and coping strategies of sexual and gender minorities in Zambia. The results of the study indicate that these populations do face several obstacles in life, which are likely the result of negative societal perceptions of sexual and gender minorities. The results also indicate that these experiences are comparable to those of developed nations. Nonetheless, the absence of protections for LGBT people in the current research setting further disadvantages them. Therefore, it is necessary to strengthen stigma reduction efforts in healthcare settings, workplaces, schools, and other areas of the community. In addition, it is necessary to establish psychosocial services tailored to the mental health needs of LGBT populations. The counseling should be tailored to their specific mental health requirements. Therefore, the type of counseling sought should focus on minority stressors, and how they contribute to poor mental health. Additionally, counseling professionals should be trained in the promotion of adaptive coping strategies. This should involve both cognitive and behavioral strategies for coping with stressful conditions. In addition, as they will be working with a criminalized population, those who provide counseling services must maintain a high level of confidentiality. The self-declaration of Zambia as a Christian nation, the political hostility and criminalization of LGBT individuals, and the general social and health structures that exclude LGBT populations create a unique circumstance that necessitates the provision of specialized services. In Zambia, mental health remains a neglected issue, even among the general population, and is not accorded the highest priority in the public health discourse. A mental health legislation was recently enacted, but additional action is required beyond this legislation.
